# Supplementary material for: Development and preliminary inter-rater reliability of the new PROOF tool to measure fidelity of problem-solving therapy for depression delivered by non-specialists in a low-resource African setting
Source: Glob Ment Health (Camb). 2025 Jul 8;12:e98. doi: 10.1017/gmh.2025.10034 (PMC12415788; doi:10.1017/gmh.2025.10034)
Supplement: Cooke et al. supplementary material [file S2054425125100344sup001.docx]

**Supplementary Material**

**Table 1. PROOF Adherence Subscale for Session 2 of Problem-solving for Depression**

|  | Interventionist Adherence Behaviour | Not done | Done |
| --- | --- | --- | --- |
| 1 | **Sets the agenda** for the session. | 0 | 1 |
| 2 | For clients with HIV and/or other chronic conditions, **reviews client’s adherence** to medication/health regimen in past week | 0 | 1 |
| 3 | **Reviews homework** from previous session | 0 | 1 |
| 4 | **Reviews client’s mood** in past week | 0 | 1 |
| 5 | **Psychoeducation about depression** symptoms and triggers | 0 | 1 |
| 6 | **Psychoeducation about negative cycle between symptoms, unhelpful behaviours and life problems** | 0 | 1 |
| 7 | **Education about problem - solving** | 0 | 1 |
| 8 | **Identifies problems** that are feasible to be worked on the therapy time frame versus ones that will take longer | 0 | 1 |
| 9 | **Problem** selected to work on **is defined AND specific** | 0 | 1 |
| 10 | **Brainstorm solutions with client** | 0 | 1 |
| 11 | **Discusses pros and cons of solutions** | 0 | 1 |
| 12 | Agrees **one solution** to implement | 0 | 1 |
| 13 | **Sets action plan** for completion of homework | 0 | 1 |
| 14 | **Schedules** next session | 0 | 1 |

##

**Table 2. PROOF Competence Subscale**

|  | Interventionist Competence Behaviour | Not done | Done |
| --- | --- | --- | --- |
| 1 | Clinician successfully **establishes rapport** with client and **demonstrates** **empathy**    0: Does not introduce themselves or attempt to make the client feel comfortable; OR dominates most of the session (e.g., talking excessively and repeatedly about what the clinician will do in the session, or about clinician’s own experiences)    1.  Demonstrates understanding and compassion, makes client feel comfortable, e.g. engages in appropriate small talk, focuses on client’s needs | 0 | 1 |
| 2 | Clinician makes good use of **open-ended questions**    0: Only or nearly exclusively uses closed and/or leading questions (e.g., “Will you? Can you?”)    1: Consistently strikes a good balance between open ended questions (e.g., “What happened How did you come to consider this? Tell me more.”) and closed questions (e.g., when clarifying information that the client has shared) | 0 | 1 |
| 3 | Clinician able to **summarise** and **reflect** to client, including on client’s emotions  0: Does not summarise to client; instead moves on quickly to next topic without reflecting back on previous one    1: Regularly and consistently summarises client’s statements in their own words (e.g., “So, what you are saying is…” and follows up with clarifying questions to ensure understanding, “Is that right? Did I understand that correctly?”) | 0 | 1 |
| 4 | Clinician tactfully **links problems identified by the client to the client’s current mental wellbeing, emotions, and/or behaviours**  0: Does not make any connection between problems and mental wellbeing, emotions, or associated behaviours, or makes the link in a tactless or stigmatising way    1: Consistently verbalises and acknowledges connection  between client's problems and current mental  wellbeing, emotions, or behaviors (e.g., normalises  depressed mood "I understand why you've been feeling  sad recently, given you cannot afford your child's  school fees.", or connects depression to challenges  engaging in HIV care "It makes sense that your sadness  makes it harder for you to remember to take your  ARVs." and/or provides client with opportunities to  process their emotions around these problems "I  understand this problem may be very difficult. Would  you like to talk about it?") | 0 | 1 |
| 5 | Clinician **gives realistic hope** about what can be achieved in therapy  0: Gives either no hope (e.g., "You will never get  better") or significant false hope (e.g., "We will  find a solution to all your problems; after this  treatment you will no longer experience thinking too  much")    1: Consistently helps client feel positive about  themselves (perhaps reminding them about a past  success) and the future, without giving false hope  (and may check clients understanding of this) | 0 | 1 |
| 6 | Clinician gives client **praise and encouragement**  0: Does not give positive feedback or praise to client Demonstrated Well  about their behaviours or completed homework    1: Praise and encouragement given consistently to the  client using locally appropriate terms to acknowledge  client has done their best/a good job | 0 | 1 |
| 7 | Clinician **collaboratively establishes homework** or other short-term goals without excessive advice-giving  0: Clinician establishes goals unilaterally or sets  all homework based on their own choices/judgments,  and/or gives excessive advice about goals or homework    1: Clinician develops goals or selects homework in  partnership with the client. Rationale is clear,  non-judgmental, and well aligned with client's  preferences. Clinician takes good account of client's  feedback if giving advice | 0 | 1 |
| 8 | Clinician delivers **clear and non-stigmatising psychoeducation** about problem-solving; and/or behavioural activation; and/or negative cycle between emotional symptoms, unhelpful behaviours, and life problems, while allowing client to show own understanding  0: Clinician educates in an unclear and/or stigmatising manner, and/or delivers all psychoeducation without checking client's  understanding of material    1: Clinician educates clearly and comprehensively, incorporating client's local world view while avoiding jargon and stigmatising statements. Clinician asks client to say what they understand about the psychoeducation | 0 | 1 |

**Table 3. Non-Specialist Adherence and Competence Ratings**

| **Adherence Subscale** | **Non-Specialist Ratings** | | | |
| --- | --- | --- | --- | --- |
| **Item Number** | **Percent Agreement** | **Gwet’s AC_1_** | **95% CI** | ***P*-value** |
| **1** | 100% | 1.00 | NaN | NaN |
| **2** | 100% | 1.00 | NaN | NaN |
| **3** | 100% | 1.00 | NaN | NaN |
| **4** | 100% | 1.00 | NaN | NaN |
| **5** | 100% | 1.00 | NaN | NaN |
| **6** | 100% | 1.00 | 1.00-1.00 | **<0.001** |
| **7** | 100% | 1.00 | NaN | NaN |
| **8** | 75% | 0.529 | –1.06-1.00 | 0.367 |
| **9** | 75% | 0.529 | –1.06-1.00 | 0.367 |
| **10** | 100% | 1.00 | NaN | NaN |
| **11** | 100% | 1.00 | NaN | NaN |
| **12** | 75% | 0.68 | –0.583-1.00 | 0.185 |
| **13** | 100% | 1.00 | NaN | NaN |
| **14** | 75% | 0.68 | –0.583-1.00 | 0.185 |
| **Average Percent Agreement** | 92.9% | | | |
| **Competence Item Number** | **Mean** | | **Percent Agreement** | |
| **1** | 1.25 | | 50% | |
| **2** | 1.25 | | 50% | |
| **3** | 1.25 | | 50% | |
| **4** | 0.50 | | 100% | |
| **5** | 1.00 | | 100% | |
| **6** | 1.00 | | 0.00% | |
| **7** | 1.5 | | 100% | |
| **8** | 1.5 | | 100% | |
| **Average Percent Agreement** | | | 68.8% | |
